# Insulin Receptor and the Kidney: Nephrocalcinosis in Patients with Recessive INSR Mutations

**DOI:** 10.1159/000366225

**Published:** 2014-10-24

**Authors:** Arabella Simpkin, Elaine Cochran, Fergus Cameron, Mehul Dattani, Martin de Bock, David B. Dunger, Gun Forsander, Tulay Guran, Julie Harris, Iona Isaac, Khalid Hussain, Robert Kleta, Catherine Peters, Velibor Tasic, Rachel Williams, Fabian Yap Kok Peng, Stephan O'Rahilly, Philipp Gorden, Robert K. Semple, Detlef Bockenhauer

**Affiliations:** ^a^UCL Institute of Child Health and Great Ormond Street Hospital for Children NHS Foundation Trust, London, UK; ^b^Diabetes, Endocrine and Obesity Branch, National Institute of Diabetes, Digestive and Kidney Diseases, Bethesda, Md., USA; ^c^Murdoch Children's Research Institute, Royal Children's Hospital, Parkville, Vic., Australia; ^d^Liggins Institute, University of Auckland, Auckland, New Zealand; ^e^The National Institute for Health Research, Cambridge Biomedical Research Centre, Cambridge, UK; ^f^Department of Paediatrics, University of Cambridge, Addenbrookes Hospital, Cambridge, UK; ^g^Queen Silvia Children's Hospital, Sahlgrenska University Hospital, Gothenburg, Sweden; ^h^Pediatric Endocrinology, Marmara University Hospital, Istanbul, Turkey; ^i^Metabolic Research Laboratories, University of Cambridge, Wellcome Trust-MRC Institute of Metabolic Science, Cambridge, UK; ^j^Department of Pediatric Nephrology, University Children's Hospital, Medical School, Skopje, Macedonia; ^k^KK Women's and Children's Hospital, Singapore

**Keywords:** Insulin receptor, INSR, Leprechaunism, Rabson-Mendenhall syndrome, Donohue syndrome, Hypercalciuria, Nephrocalcinosis

## Abstract

**Background/Aims:**

Donohue and Rabson-Mendenhall syndrome are rare autosomal recessive disorders caused by mutations in the insulin receptor gene, *INSR*. Phenotypic features include extreme insulin resistance, linear growth retardation, paucity of fat and muscle, and soft tissue overgrowth. The insulin receptor is also expressed in the kidney, where animal data suggest it plays a role in glomerular function and blood pressure (BP) regulation, yet such a role in the human kidney is untested. Patients with biallelic INSR mutations provide a rare opportunity to ascertain its role in man.

**Methods:**

Retrospective review of patients with INSR mutations. Data for BP, renal imaging, plasma creatinine and electrolyte levels, as well as urine protein, albumin and calcium excretion were sought from the treating clinicians.

**Results:**

From 33 patients with INSR mutations, data were available for 17 patients. Plasma creatinine was low (mean ± SD: 25 ± 9 μmol/l) and mean plasma electrolyte concentrations were within the normal range (n = 13). Systolic BP ranged between the 18th and 91st percentile for age, sex, height and weight (n = 9; mean ± SD: 49 ± 24). Twenty-four-hour urinary calcium data were available from 10 patients and revealed hypercalciuria in all (mean ± SD: 0.32 ± 0.17 mmol/kg/day; normal <0.1). Nephrocalcinosis was present in all patients (n = 17). Urinary albumin excretion (n = 7) ranged from 4.3-122.5 μg/min (mean ± SD: 32.4 ± 41.0 μg/min; normal <20).

**Conclusions:**

INSR dysfunction is associated with hypercalciuria and nephrocalcinosis. No other consistent abnormality of renal function was noted. Normotension and stable glomerular function with only moderate proteinuria is in contrast to genetically modified mice who have elevated BP and progressive diabetic nephropathy.

## Introduction

Donohue syndrome (DS) and Rabson-Mendenhall syndrome (RMS) are rare autosomal recessive disorders. Initially described as distinct entities, they have subsequently been found to be caused by mutations in the insulin receptor gene (INSR). They are characterized by intrauterine and postnatal growth restriction, paucity of adipose tissue, overgrowth of many soft tissues including skin, hair and teeth, and characteristically coarse facial features, as well as severely abnormal glucose homeostasis and extreme insulin resistance. DS denotes the more severe end of the symptom spectrum and is often associated with death in the first year of life. It was first described by Donohue in 1948 [1], and termed ‘leprechaunism’ in 1954 [2]. RMS, originally described in 1956 [3], generally is taken to encompass slightly less severely affected patients, although the distinction between DS and RMS is not strictly defined. RMS also features linear growth impairment, soft tissue overgrowth, and coarse features. The severe insulin resistance leads to hyperinsulinism with pancreatic β-cell decompensation, diabetes, hyperglycemia and ultimately ketoacidosis, usually towards the end of the first or in the second decade, as well as hyperandrogenism. Early mortality due to advanced complications of diabetes is common [4]. A more common but less severe phenotype, often called type A insulin resistance, features insulin-resistant diabetes and hyperandrogenism that usually is identified peri- or postpubertally. In most causes this is accounted for by dominant negative heterozygous mutations in the tyrosine kinase domain of the receptor, however a minority of cases are caused by biallelic, presumably less deleterious, α-subunit mutations [4].

The INSR is expressed throughout the nephron [5,6], but little is known about its precise role. In vivo perfusion experiments in rabbits showed a positive effect of insulin on proximal tubular sodium reabsorption [7], yet the absence of insulin signaling in mice with kidney-specific deletion of INSR is also associated with enhanced sodium reabsorption and consequent elevated blood pressure (BP) [8]. Podocyte-specific deletion of the receptor in mice has been reported to lead to direct remodeling of the actin cytoskeleton with consequent proteinuria and features of diabetic nephropathy [9]. Recently, INSR has also been implicated in magnesium homoeostasis [10,11]. Patients with impaired INSR function provide a unique opportunity to study the role of this receptor in human kidney. So far, only case reports and small case series exist in the literature, reporting an association with medullary sponge kidney [12,13], and a Bartter-like phenotype [14]. In order to get a better understanding, we performed a medical note review of the renal features of INSR dysfunction in the largest series of patients reported so far.

## Materials and Methods

Subjects with severe insulin resistance and a clinical diagnosis of DS or RMS were recruited for research studies with informed consent in line with procedures approved by either the local research ethics committee in Cambridge, UK, or the institutional review board of the National Institute of Diabetes and Digestive and Kidney Diseases, USA.

Information on BP, renal ultrasound, urine output, plasma creatinine and electrolytes, as well as urine protein, albumin and calcium excretion were sought for those patients with confirmed *INSR* mutations. If multiple datasets were available from the same patient, the most recent and most complete dataset was used.

## Results

From a research database of 33 patients with *INSR* mutations, data on renal features were available for 17 patients from the clinicians contacted, although the complete dataset was only available for 8 cases (table 1).

### General Characteristics and INSR Mutations

Age at last follow-up varied between 4 months and 24 years. Seven patients had a clinical diagnosis of DS and 10 of RMS. Mutations and/or clinical data not pertaining to kidney function have been previously reported for 9 of the patients [4,15,16,17,18,19]. Previously unreported mutations include four altering conserved residues in the L1 domain of the α-subunit (p.L109R, p.R114W, p.G84Q, p.V66A), and one affecting a conserved residue in the extracellular β-subunit (p.R899Q).

### Blood Pressure

Due to the discordant animal model results with respect to insulin signaling and BP [7,8], we first reviewed BPs of the patients. As systolic BP is more reliably measured, especially in small children, we concentrated on these and translated them into centile values, based on age, sex, height and weight. BP data were available from 9 children and the mean centile was the 49th, albeit with a wide range (18th-91st).

### Glomerular Function

Data from genetically modified mice suggested a critical role for Insr in podocyte function [9] and we therefore assessed glomerular filtration rate, protein and albuminuria. Formal GFR measurements were not available, but plasma creatinine values were in the low-normal range for age in the 13 patients with available data. Six patients had proteinuria quantified via a 24-hour urine collection with mean proteinuria being 10.1 mg/m^2^/h (range 0.48-27.16) and thus just above the upper limit of normal (<4 mg/m^2^/h). Similarly, 24-hour data on albuminuria were available from 7 patients with mean albumin excretion being 32.4 μg/min (range 4.3-122.5), thus again just above the upper limit of normal (<20 μg/min).

### Plasma Electrolytes, Renin and Aldosterone, Parathyroid Hormone and Vitamin D

Plasma electrolytes were generally in the normal range, although mean plasma potassium was in the low-normal range at 3.78 mmol/l, with 3 patients being hypokalemic (<3.5 mmol/l; table 1). Similarly, mean plasma chloride level was in the low-normal range at 101 mmol/l, with 3 patients having values below the norm. Mean plasma bicarbonate concentration was in the high normal range at 26 mmol/l. Hypokalemic, hypochloremic alkalosis suggests activation of the renin-aldosterone system. Unfortunately, renin and aldosterone values were available in only 4 patients and showed slightly elevated renin activity [13 (patient 12), 37 (patient 13) and 8.7 (patient 14) pmol/ml/h and 162 mIU/l (patient 16; normal <40)], yet normal or even suppressed aldosterone values [400 (patient 12), <160 (patient 13), 170 (patient 14) and 320 (patient 16) pmol/l; normal <800]. Thus, the aldosterone/renin ratio was low.

Mean total plasma calcium level (n = 15) was normal at 2.28 mmol/l. Parathyroid hormone levels (n = 10) were in the low-normal range in all with a mean ± SD of 2.3 ± 0.7 pmol/l (normal 0.3-5.9). 25-OH vitamin D levels were available in 9 patients and normal in all, ranging from 25 to 128 nmol/l (mean ± SD: 73 ± 31; normal 25-200). Plasma magnesium levels were in the low-normal range with a mean of 0.73 mmol/l (n = 13), with 5 patients being hypomagnesemic (table 1).

### Urine Volume

Data on 24-hour urine volume were available in 8 patients and urine output varied between 1.3 and 8.3 (mean ± SD: 3.8 ± 1.8) ml/kg/h.

### Urine Calcium Excretion

Twenty-four-hour urine calcium excretion was quantified in 10 patients and ranged from 0.12 to 0.68 (mean ± SD: 0.32 ± 0.17) mmol/kg/day, and was thus above the upper limit of normal (0.1 mmol/kg/day) in all.

### Nephrocalcinosis

**Fig. 1:**
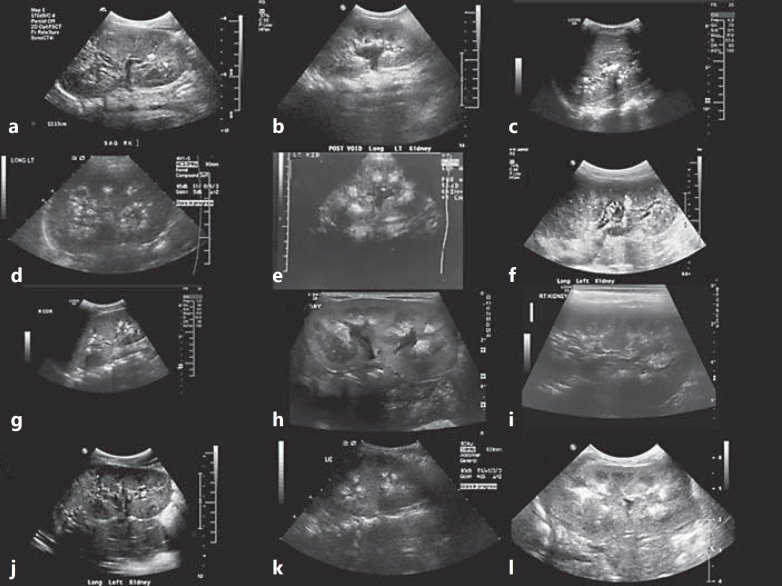
Representative ultrasound images detailing nephrocalcinosis. Shown are ultrasound images of kidneys from patient 1, right kidney, age 10 years (**a**), patient 3, age 16 years (**b**), patient 4, left kidney, age 24 years (**c**), patient 8, left kidney, age 12 years (**d**), patient 5, age 9 months (**e**), patient 9, left kidney, age 14 years (**f**), patient 10, right kidney, age 17 years (**g**), patient 12, left kidney, age 6 months (**h**), patient 13, right kidney, age 8 years (**I**), patient 14, left kidney, age 13 years (**j**), patient 15, left kidney, age 13 years (**k**), and patient 16, left kidney, age 3 months (**l**). Note the varying pattern of nephrocalcinosis by ultrasound, ranging from isolated echodensities to a generalized punctuate pattern to medullary nephrocalcinosis.

**Fig. 2:**
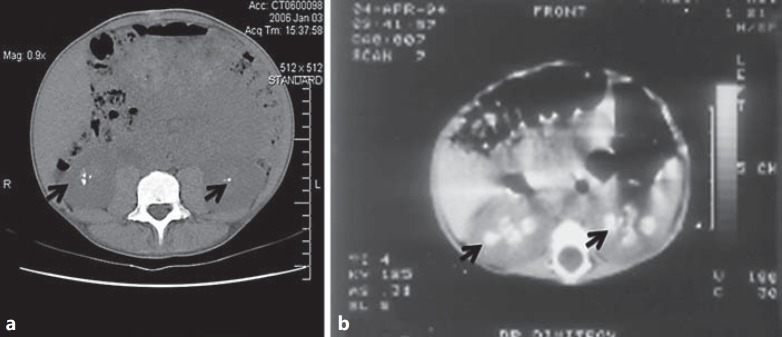
Representative CT images detailing nephrocalcinosis. Shown are abdominal CT images from patient 4, age 24 years (**a**) and patient 5, age 9 months (**b**). Note the calcium densities in the kidneys (arrows).

Data relevant to nephrocalcinosis were available in all patients. All had renal ultrasound imaging reported as consistent with nephrocalcinosis. This was confirmed by CT in a further 2 patients (fig. 1, 2). In 1 patient (patient 12), nephrocalcinosis was even reported on an antenatal ultrasound.

## Discussion

We report the renal features in the largest series of patients with confirmed *INSR* mutations to date in order to illuminate the role of the INSR in the kidney. Interestingly, key features identified in this series are in contrast to some observations made in genetically modified animals, suggesting that there may be species-specific differences in INSR function in the kidney. Kidney-specific knockout of Insr in mice resulted in increased BP and impaired natriuresis suggesting that INSR is involved in BP regulation by enhancing sodium excretion [8]. However, our data provide no evidence for enhanced sodium absorption in our patients as BPs were normal. Moreover, primary sodium retention would be expected to result in suppressed plasma renin activity yet this was actually slightly elevated in the 4 patients with these data available, albeit without concomitant hyperaldosteronism.

Recently, the insulin receptor was reported to be critically important for glomerular function in mice: podocyte-specific deletion of the *Insr* gene is associated with albuminuria, glomerulosclerosis and chronic kidney disease [9]. A biopsy in a patient with DS also showed features of diabetic nephropathy [20]. Moreover, in a long-term follow-up (up to 30 years) report of 11 patients with INSR mutations (8 with type A insulin resistance and 3 with RMS), nephropathy occurred in 5, including 1 of the RMS patients who had proteinuria of up to 600 mg/day [4]. The low-grade proteinuria observed in our patients is in keeping with these findings, but there was no evidence of impaired kidney function at least as assessed by plasma creatinine. Whilst we recognize the imperfect nature of creatinine as a marker of glomerular filtration in these patients due to their low muscle mass, values remained very low up to 24 years of age. Kidney biopsies had not been performed in any of our patients, thus we are unable to comment on glomerulosclerosis, but it was not reported in the autopsy findings of the original publications by Donohue [1,2]. This may reflect the age difference between the autopsy patients (infants) and the case report (10 years).

Differences between mice and men have also been observed for other important aspects of the phenotype of insulin receptor deficiency. Most strikingly, *Insr* null mice die soon after birth of diabetic ketoacidosis [21], whereas humans with no functional insulin receptors seem protected from ketoacidosis at least in the early years of life [22,23].

The key renal features identified in our patients are hypercalciuria and nephrocalcinosis. This fits well with original autopsy findings of intratubular calcium deposits reported by Donohue [1,2]. Interestingly, in 1 patient (patient 12) in whom antenatal ultrasound data were available, nephrocalcinosis was already reported before birth. Nephrocalcinosis has also been reported in other patients with DS [14,24]. The hypercalciuria in our patients was assessed by the gold standard of 24-hour urine collection, as the appropriateness of the calcium/creatinine ratio – commonly performed in children – is questionable in these patients: they are small for their age and have low apparent muscle mass, reflected in low plasma creatinine values with consequent lower than normal creatinine excretion.

The finding of hypercalciuria with INSR dysfunction fits well with the long-recognized hypercalciuria seen in patients with diabetes. The reversibility of this with insulin treatment also supports a role for INSR in renal calcium handling [25].

Nevertheless, a key question arising from our observations is to what degree the renal manifestations result from a direct effect of the insulin receptor on renal function or merely reflect the altered metabolism and glucose handling in this condition or signaling through alternative pathways, such as the IGF receptor.

Several lines of evidence support a primary role of the insulin receptor in renal calcium handling. We observed antenatal nephrocalcinosis in the 1 patient with available data, despite the ‘clamping’ of glucose levels in utero by the placenta. Although bone buffering of (keto)acidosis is thought to increase calcium release from bone with consequent hypercalciuria [26], the patients described here were not acidotic at the time the calcium excretion was measured. In fact, the trend for increased plasma bicarbonate levels rather suggests metabolic alkalosis. Patients with the milder ‘type A’ form of INSR dysfunction who have hyperinsulinemic diabetes mellitus may develop features of diabetic nephropathy, yet have not been reported to show nephrocalcinosis even when observed for many years [4].

Obviously, the observational data provided here do not establish an underlying mechanism and the localization of the tubular impairment in calcium reabsorption can only be speculated upon: the majority of filtered calcium is reabsorbed passively in the proximal tubule [27], via a yet unidentified molecular pathway. There was no other evidence of proximal tubular dysfunction: low molecular weight protein excretion, the most sensitive indicator of proximal tubular dysfunction [28,] was normal, as was renal phosphate and glucose handling (data not shown). This fits with animal data, as specific knockout of Insr in the proximal tubule also does not result in any tubular abnormalities in mice [29]. Approximately 20% of calcium is reabsorbed in the thick ascending limb of Henle's loop (TAL) through a paracellular pathway involving claudin 16 and 19 [30]. This pathway is also used for the reabsorption of magnesium and sodium. Thus, defects in it are associated with renal magnesium loss and hypomagnesaemia. As this pathway is driven by and involved in sodium chloride reabsorption in TAL, polyuria and hypokalemic, hypochloremic metabolic alkalosis are often associated [31]. This would fit with the trend for electrolytes identified here, as well as with the reported association with a Bartter-like phenotype [14]. Approximately 15% of filtered calcium is reabsorbed in the distal convoluted tubule and connecting tubule via a transcellular pathway involving TRPV5 and TRPV6 [32]. So far, no human disease has been associated with dysfunction of these channels, so the phenotype in humans is unclear. Given the expression of INSR along the entire nephron, it could, of course, also be a combination of altered calcium handling in all these segments.

A recent report details an association of RMS with medullary sponge kidney [13]. We are unable to confirm or refute this association in our series, as medullary sponge kidney is a diagnosis made by the typical appearance of the kidneys on an intravenous pyelogram and none of our patients had this examination performed.

## Conclusion

Mutations in *INSR* in man lead to abnormalities in kidney function, most prominently to hypercalciuria and nephrocalcinosis, suggesting an important role for INSR in renal calcium handling. In addition, there was a trend towards hypokalemic, hypochloremic alkalosis and hypomagnesemia. Further investigations are needed to dissect the underlying mechanisms.

Our findings are in contrast to reports from mice, where specific deletion of the insulin receptor is associated with marked albuminuria and histological features reminiscent of diabetic nephropathy [9], as well as enhanced sodium reabsorption [8]. These discrepant results suggest that there may be differences between men and mice when it comes to the function of the insulin receptor in the kidney.

## Disclosure Statement

The authors have no competing financial interests.

## Figures and Tables

**Table 1:** Overview of kidney-related renal features

	Identifier																
	P1	P2	P3	P4	P5	P6	P7	P8	P9	P10	P11	P12	P13	P14	P15	P16	P17
Age at evaluation, years	11	0.3	22	24	0.9	5	10	13	13	16	0.1	0.6	10	12	13	6	0.3

Clinical diagnosis	RMS	DS	RMS	RMS	DS	DS	RMS	RMS	RMS	RMS	DS	DS	DS	RMS	RMS	RMS	DS

INSR mutation(s)	P.L109R/g.26624_26625delA	P-N431D (H)	P-P193L (H)	P-P193L (H)	P-C274Y/P-R1174W	P-R114W (H)	P-R899Q/IVS18-1G>T	P-L460E/P-L109R	P.I119M (H)	P-S608L/del ex9-10	P-Q521X (H)	P-G84Q (H)	P.I119M/P-R1039X	P-Y30X/P-E238L	R1092Q (H)	P-V66A/del ex18	IVS7 + ldelT/p.325_331 del

Weight, kg	25.2	2.0	30.9	35.0	3.7	–	24.4	36.2	37.9	38.2	1.44	3.0	19.0	35.8	12.4	12.5	2.8

Plasma creatinine,	μmol/l	13.9	30	12.8	19.4	20	20	48	20	19	13.3	20.5	12	22	22	23	8	–

Plasma total	calcium, mmol/1	2.34	2.78	2.3*	2.23*	2.51*	2.36	2.72	2.09	2.21	2.28	2.65	2.27	2.43	2.26	2.43*	2.26	–

Plasma potassium, mmol/1	3.6	4.8	4.0	4.2	3.2	3.8	3.9	3.0	–	3.9	3.8	2.8	3.6	3.8	4.2	3.5	–

Plasma chloride, mmol/1	103	96	103	104	93	–	102	102	102	104	101	98	104	103	92	–	–

Plasma tCO_2_, mmol/1	26	28	26	27	28	–	–	23	–	25	–	29	27	27	29	19	–

Plasma magnesium, mmol/1	0.71	1.0	0.75	0.78	0.67	–	0.8	0.65	–	0.59	0.5	0.95	0.67	–	0.95	0.64	–

Urinary calcium excretion^1^, mmol/kg/24 h	0.14		0.45	0.12	0.41			0.18	0.29	0.35			0.68	0.21		0.38	

Presence of nephrocalcinosis	yes	yes	yes	yes	yes	yes	yes	yes	yes	yes	yes	yes	yes	yes	yes	yes	yes

Not all data were available for all patients. Plasma calcium values are not corrected for plasma albumin, unless indicated by*, as albumin levels were not available in all. Note that nephrocalcinosis was noted in all patients. Mutation annotation is according to the mature isoform B of the INSR, based on reference sequence P062134.4. Note that amino acids are numbered from the first codon of the mature receptor, which means that they are 27 less than the numbering of the reference sequence.

(H) = Homozygous.
